# Oxidative Stress Contributes to Cytoskeletal Protein Degradation of *Esox lucius* through Activation of Mitochondrial Apoptosis during Postmortem Storage

**DOI:** 10.3390/foods11091308

**Published:** 2022-04-29

**Authors:** Xue Li, Pingping Liu, Yunfeng Zhao, Lianfu Zhang, Jian Zhang

**Affiliations:** 1 School of Food Science and Technology, Shihezi University, Shihezi 832003, China; 18152082056@163.com (X.L.); liupp0222@163.com (P.L.); yunfeng@shzu.com.edu.cn (Y.Z.); lianfu@jiangnan.edu.cn (L.Z.); 2 State Key Laboratory of Food Science and Technology, School of Food Science and Technology, Collaborative Innovation Center of Food Safety and Quality Control in Jiangsu Province, Jiangnan University, Wuxi 214122, China

**Keywords:** reactive oxygen species, caspases, mitochondrial apoptosis, cytochrome c, myofibril protein

## Abstract

This study investigated the role of oxidative stress in the mitochondrial apoptotic pathways and structural protein degradation of fish during postmortem storage by measuring oxidative stress levels, mitochondrial antioxidant enzyme activity, mitochondrial dysfunction, apoptotic factors, and structural protein degradation (*n* = 3). The results revealed that reactive oxygen species (ROS) increased gradually within the first 12 h and then decreased (*p* < 0.05) in mitochondria. Lipid peroxidation was increased, and superoxide dismutase, catalase, and glutathione peroxidase activities were decreased in mitochondria (*p* < 0.05). Furthermore, oxidative stress induced mitochondrial membrane opening, mitochondrial swelling, as well as the depolarization of mitochondrial potential. This led to an increase in the release of cytochrome c from mitochondria and caspase-3 activation. Ultimately, oxidative stress promoted small protein degradation (troponin-T and desmin) and induced myofibril susceptibility to proteolysis. These observations confirmed that oxidative stress mediated the activation of mitochondrial apoptotic factors-promoted protein degradation, initiating the deterioration of fish muscle through the mitochondrial apoptotic pathway.

## 1. Introduction

Numerous fish species offer high nutritional value and have become popular because of their attractive taste. Fish texture is an important economic attribute, as it influences consumer preference and acceptability [1]. Northern Pike (*Esox Lucius*) is one of the most important socio-economic freshwater fish species, providing various nutrients that are essential for human health. Meanwhile, due to its low-fat characteristics, *Esox Lucius* is highly attractive to consumers [2,3]. The fatty acids in the raw fish fillets are mainly palmitic acid (42.6%), oleic acid (12.1%) and stearic acid (11.1%) [4]. However, the fish texture is susceptible to deterioration during postmortem storage. This phenomenon is the result of active biochemical and microbial activities, especially endogenous proteolytic enzymes [5]. Apoptosis can regulate protein proteolysis, as a crucial biochemical change. An increasing number of studies indicate the central role of the apoptosis proteolytic system, and it is proposed that apoptosis occurs in the myofibrillar protein degradation of fish during storage [6].

Apoptosis refers to active, gene-controlled cell death that is highly regulated in metabolic processes. It is characterized by cell shrinkage, the formation of apoptotic bodies, and DNA fragmentation. After bleeding, oxygen absence and nutrient deprivation occur in skeletal muscle that might cause the intrinsic pathway apoptosis affected by mitochondrial dysfunction in the muscle [7]. The mitochondrial pathway is considered a possible mechanism for skeletal muscle tenderness postmortem, involving cytochrome c release and caspase-3 activation. During the apoptotic process, mitochondria, as crucial gateway controllers, are affected by postmortem changes regulating apoptosis. Apoptotic stimuli factors that disrupt the apoptotic process are rarely reported in fish deterioration, due to the complexity of fish texture. Therefore, further study on the apoptotic activation affecting fish protein degradation during storage is needed.

According to previous studies, once cytochrome c is released into the cytoplasm, it can rapidly form apoptosomes by binding with the apoptotic activating factor (Apaf-1) in the presence of d-ATP. Apoptosomes activate pro-caspase-9 and the effector capsase-3 downstream [8]. This process will lead to mitochondrial-dysfunction-induced apoptosis and the activation of a cascade of caspases. Caspases are the main executors in apoptosis. Activated caspase-3 degrades key myofibrillar proteins and the structure of muscles [9]. It has been suggested that caspase-3 can degrade structure proteins, and the activation of caspase-3 is mediated by the release of cytochrome c in the early stage [10]. Mitochondrial membrane permeability transition pores (MPTPs) are regarded as major passageways for the release of cytochrome c in mitochondria; the release is accompanied by the depolarization of mitochondrial membrane potential (MMP) and mitochondrial dysfunction [11].

Reactive oxygen species (ROS) are known as the major stimulators and examples of molecules participating in cell apoptosis. Mitochondria are major producers of ROS in cells, making them susceptible to oxidative stress. Several studies have demonstrated that ROS accumulation can induce the peroxidation of polyunsaturated fatty acids in the mitochondrial membrane. This leads to mitochondrial damage, as evidenced by the collapse of MMP, mitochondrial swelling, and oxidative cytochrome c production [12]. Mitochondria are protected against mitochondrial ROS overproduction via endogenous antioxidant systems [13]. To limit oxidative damage, superoxide dismutase (SOD) can catalyze the transformation of O_2_^−^ to H_2_O_2_, which can be converted to H_2_O and O_2_ when coordinated with catalase (CAT). Reduced glutathione (GSH) also plays a crucial role in scavenging primary ROS and protecting the cell against damage caused by free radicals [14]. A substantial amount of evidence has shown that ROS can mediate apoptosis during the postmortem storage of muscles. Hydrogen peroxide is highly reactive and is well suited for evaluating the degree of oxidative stress in various cells [15,16]. However, due to the complex mediation mechanism, the ability of ROS to regulate the activation of apoptotic caspase and its contribution to fish protein degradation through the mitochondrial apoptotic pathway require further investigation.

Hydrogen peroxide is highly reactive and is well suited for evaluating the degree of oxidative stress in various cells and inducing cell apoptosis [17]. In the present study, hydrogen peroxide was used to explore the effect of oxidative stress on mitochondrial-dysfunction-induced apoptosis, through the induction of mitochondrial lipid peroxidation, mitochondrial membrane swelling and the release of cytochrome c. Moreover, this study also aimed to elucidate the correlation between the activation of the mitochondrial apoptotic pathway mediated by ROS and fish softening by evaluating the changes in caspase-3/9 activation, the redox state of cytochrome c, and cytoskeletal protein degradation in fish muscles during postmortem.

## 2. Materials and Methods

### 2.1. Sampling

Esox lucius (0.9–1.1 kg, 40–45 cm, *n* = 15) was purchased from a local supermarket in Shihezi (Xinjiang, China) and transported to the laboratory alive in an oxygenated container, stunned instantly by a blow to the head with a wooden stick [18]. The fish were randomly divided into three groups, and each group included five samples. The dorsal muscles were obtained immediately and cut into 60 pieces (approximately 30 g each). Each sample in the group was injected with 20 mM NAC (Beyotime Biotechnology, Shanghai, China) at a ratio of 10:1 (*w*/*v*). Surplus samples in group were treated with 20 mM H_2_O_2_ at the same ratio, which was based on a previous study [19,20]. The control group received no treatments. In addition, the 60 pieces were divided equally into the same three treatment groups and three replicates at each storage point for future analysis, where the remaining samples were reserved. The samples were stored at 4 °C and aged for 2, 6, 12, 24, 72, and 120 h. At the end of each storage period, the samples were individually obtained, flash frozen in liquid nitrogen, and stored at −80 °C until use for biochemical analysis.

### 2.2. Myofibrillar Protein (MP) Extraction

The myofibrillar protein was extracted via the previous method [21], with minor modifications. Briefly, the dorsal muscles were homogenized with pre-chilled deionized water (30 mL) using a homogenizer at 10,000 rpm for 1 min (Scientz Co., Ningbo, China). The mixture was centrifuged at 8000 g for 15 min at 4 °C (Thermo Fisher Technologies, Waltham, MA, USA). The supernatant was discarded, and the precipitates were re-suspended in 5 mM NaCl. The mixture was centrifuged according to the above procedure. The collected precipitates were combined with 20 mM Tris-HCl buffer (pH 7.0) containing 0.6 M NaCl to dissolve proteins. Next, the sample was centrifuged again at 8000 g for 15 min at 4 °C, and the pellets were obtained. Finally, the MP pellets were dissolved in 0.6 M NaCl solution and filtered with gauze to remove connective tissues. Protein concentration was determined via the Biuret assay.

### 2.3. Mitochondrial Protein Extraction

The mitochondrial protein was obtained via a previously described method, with slight modifications [22]. One gram of minced muscle was homogenized with pre-cooled extraction solution (250 mmol/L sucrose, 20 mmol/L HEPES, 10 mmol/L KCl, 1.5 mmol/L MgCl, 1 mmol/L EDTA, 1 mmol/L EGTA, 1 mmol/L DTT, pH 7.4). Then, the mixture was centrifuged at 800 g for 15 min at 4 °C. The collected supernatant (S1) was centrifuged at 20,000 g for 20 min at 4 °C. The supernatant (S2) resulting from the above procedure was also centrifuged at 20,000 g for 20 min at 4 °C to remove residual mitochondria. Finally, the mitochondrial pellets (P2) were resuspended in the extraction solution. The resulting supernatant was a cytosolic protein and used to determine the expression of cytochrome c.

#### 2.3.1. Measurement of Mitochondrial ROS

Mitochondrial ROS generation was analyzed via the modified method [23], using a fluorescent probe (DCFH-DA, New Jersey, USA). Mitochondrial pellets (0.1 mg protein) were incubated and combined with pre-cooled 50 mM phosphate buffer (pH 7.4), resulting in a final volume of 3 mL and DCFH-DA concentration of 5 μM. The mixture was pre-incubated at 37 °C for 15 min to allow DCFH-DA to penetrate the membranes. To remove excess fluorescent indicator, the mixed solution was centrifuged at 12,500 g for 8 min at 4 °C. Subsequently, the pellets were resuspended in 50 mM phosphate buffer. The changes in fluorescence intensity were measured using a fluorescence spectrophotometer (970 CRT, Shanghai Precision Instrument Co., Ltd., Shanghai, China) at 488 nm for excitation and 525 nm for emission.

#### 2.3.2. Determination of Mitochondrial Antioxidant Enzyme Activity and MDA Levels

The activities of CAT, GSH-Px, SOD and MDA were assessed with commercial kits, according to the manufacturer’s instructions (Nanjing Jiancheng Bioengineering Institute, Nanjing, China). Briefly, CAT activity was indirectly detected by monitoring H_2_O_2_ consumption. One unit of CAT activity is equal to the amount of enzyme that will consume 1 μmol H_2_O_2_ per 1 mg tissue proteins in 1 s, which was measured by changes in absorbance at 405 nm via ultraviolet spectrophotometry. GSH-Px activity was assayed to evaluate the enzymatic reaction in mitochondrial pellets. The changes in the absorbance of oxidized GSH were measured at 412 nm via ultraviolet spectrophotometry. The SOD activity was evaluated via the auto-oxidation of hydroxylamine, and one unit of enzyme activity is defined as the amount of enzyme required to restrain the dye at 50% inhibition in a total volume of 1 mL mixture solution per 1 mg proteins. The level of mitochondrial liquid peroxidation, in terms of malondialdehyde (MDA) content were measured via spectrophotometry at 532 nm.

#### 2.3.3. Detection of MPTP Opening and MMP

The opening of MPTP in mitochondria reflecting the changes in absorbance was estimated by a modification procedure [24]. The separated mitochondrial pellets were suspended in ice-cold MPTP test medium containing 230 mM mannitol, 70 mM sucrose and 3.0 mM Hepes (pH 7.4). Subsequently, the mitochondrial protein was adjusted to 0.3 mg/mL and incubated with the test medium at a ratio of 1:9 (*v*/*v*) for 3 min at 25 °C. The absorbance was immediately determined using a UV spectrophotometer at 540 nm.

The MMP was evaluated using commercial assay kits (Solarbio, Beijing, China), according to the manufacturer’s instructions. Briefly, isolated mitochondrial pellets (100 µg) were mixed with JC-1 dye working solution (9-fold volume), incubated for 30 min at 37 °C. Subsequently, the fluorescence intensity was measured using a fluorescence spectrophotometer (970 CRT, Shanghai Precision Instrument Co., Ltd., Shanghai, China) with the following parameters: EX = 525 nm and Em = 590 nm for J-aggregates (Red); EX = 490 nm and Em = 530 nm for monomers (Green). The ratio of red/green fluorescence intensity was used to denote the level of mitochondrial membrane depolarization potential.

#### 2.3.4. Measurement of Mitochondrial Swelling

The mitochondrial proteins were extracted as described in Section 2.3. Mitochondrial swelling was monitored via a previously described method [25], with few modifications. Briefly, mitochondrial proteins (0.5 mg/mL) were mixed with an incubation medium containing 0.5 mM FeSO_4_ and 0.5 mM ascorbic acid. The mixture was incubated for 15 min at 37 °C. The absorbance of the mixture was measured at 520 nm using a Cary 50 spectrophotometer (Shanghai Spectrum Instrument Co., Ltd., Shanghai, China) to determine mitochondrial swelling.

### 2.4. Ultrastructural Observation of Mitochondria

The isolated mitochondria were visualized via transmission electron microscope (TEM), according to the method described previously [26]. Based on the isolation method (Section 2.3), the separated mitochondrial pellets were immobilized in 2.5% glutaraldehyde overnight. Then, the sample was rinsed in phosphate buffer three times and post-fixed in 1% tetroxide osmium for 1 h. The sample was dehydrated in an ethanol series for 5 min at each step. Subsequently, 100% propylene oxide replaced the ethanol in the sample (15-min incubation). After being infiltrated and embedded with resin and propylene oxide, the sample was cured at 35 °C for 5 h, 60 °C for 5 h, and 70 °C for 9 h. Semi-thin sections were cut and stained with uranyl acetate and lead citrate for estimation via TEM (HT7700, Hitachi Ltd., Tokyo, Japan).

### 2.5. SDS-PAGE and Immunoblot Analysis

Samples containing myofibrillar proteins and cytochrome c were boiled with 2× loading buffer (0.5 M Tris, 50% glycerol, 20% SDS, pH 6.8) for 5 min and stored at −80 °C until loading. Equal amounts of protein were run on 15% (for cytochrome c) and 5% separation gel (for other proteins). After electrophoresis, the target proteins were transferred to PVDF membranes with a semi-dry transfer apparatus (Bio-Rad Laboratories, Hercules, CA, USA). The membranes were blocked with TBST buffer containing 5% non-fat milk for 2 h at room temperature. Then, the membranes were soaked and underwent 24 h of incubation with primary antibodies at 4 °C: anti-actin mouse monoclonal antibody (Sigma-Aldrich, St. Louis, MO, USA; 3E9 at 1:2000); anti-desmin mouse monoclonal antibody (Abcam, Cambridge, UK; ab8470 at 1:4000); anti-troponin-T mouse monoclonal antibody (Abcam, Cambridge, UK; ab10214 at 1:4000); anti-cytochrome c mouse monoclonal antibody (Abcam, Cambridge, UK; ab110325 at 1:2000); anti-β-actin mouse monoclonal antibody (Sigma-Aldrich, MO, USA; A3584 at 1:4000). After washing three times with TBST for 10 min, the membranes were probed with appropriate goat anti-mouse IgG H&L (HRP) conjugate secondary antibody (Sigma-Aldrich, MO, USA; AP308P at 1:10,000) for 1 h. Finally, the membranes were washed with TBST, and the intensity of the immunoblot bands was evaluated using Quantity One software (Bio-Rad Laboratories, Hercules, CA, USA).

### 2.6. Analysis of Caspases and Cytochrome C Redox State of Mitochondria

The activities of caspase-3 and caspase-9 were analyzed using determination assay kits (Beyotime Biotechnology, Shanghai, China), according to the manufacturer’s instructions. One unit is calculated as the amount of enzyme required to catalyze 1.0 nmol Ac-DEVD-pNA at 37 °C per hour. The mitochondrial cytochrome c redox state was determined by following a modified method [9]. Briefly, the concentration of cytochrome c extract (Section 2.3) was measured according to the Biuret method. The changes in cytochrome c redox state were determined by monitoring the changes in absorbance at 550 nm minus that at 535 nm, normalized as the concentration of total protein.

### 2.7. Statistical Analysis

Each experiment was performed three times. The results are expressed as the mean ± standard deviation (SD) and analyzed by Duncan’s multiple-range test using SPSS software (version 19.0). Differences were regarded as statistically significant at *p* < 0.05.

## 3. Results and Discussion

### 3.1. ROS Induced Mitochondrial Oxidative Stress

**Figure 1:**
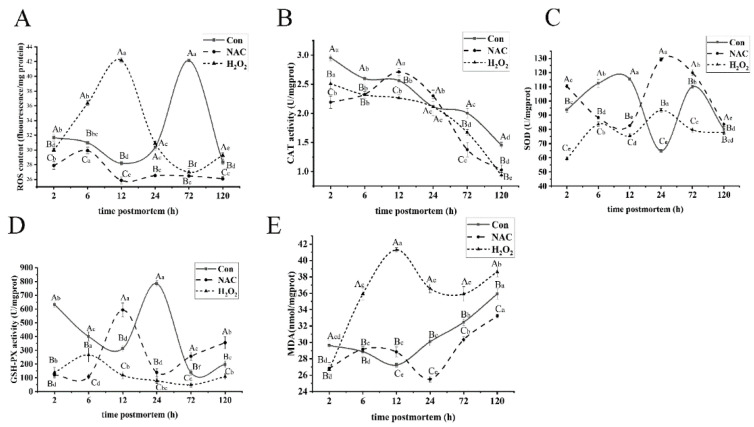
The changes in mitochondrial ROS (**A**), mitochondrial antioxidative enzyme activities of CAT (**B**), SOD (**C**), and GSH-PX (**D**) and mitochondrial MDA concentration (**E**) in the NAC, H_2_O_2_, and control groups when stored at 4 °C for 2, 6, 12, 24, 72, and 120 h. Duncan’s multiple-range test was used to analyze the differences in the three groups at different times, represented by lowercase letters (*p* < 0.05). The differences among the three groups at the same time point were analyzed by Duncan’s multiple-range test, represented by capital letters. The error bars indicate the standard error of the mean (*n* = 3).

Mitochondrial ROS generation is inevitable in postmortem aging. It is crucial for sustaining redox homeostasis in muscle cells and maintaining an ROS level below the threshold. When antioxidant homeostasis is disrupted, ROS accumulation will damage cellular biomolecules and interfere with their biological functions [27]. In order to explore the degree of mitochondrial oxidative stress, the ROS level was evaluated upon treatment with H_2_O_2_ and NAC during the postmortem storage of fish (Figure 1A). The ROS content in the H_2_O_2_ group increased (*p* < 0.05) at 2–12 h postmortem, and it was much higher (*p* < 0.05) than those of the control and NAC groups. However, the ROS level in the NAC group was significantly inhibited during storage, compared with the control and H_2_O_2_ groups (*p* < 0.05). Antioxidant enzymes are important protectors against ROS that translated O_2_^−^ and H_2_O_2_ into O_2_ and H_2_O with weak oxidizing capacity. CAT, SOD, and GSH-Px are among the most effective enzymes in protecting cells against oxidative stress [28]. The CAT activity (Figure 1B) in the H_2_O_2_ treatment group was significantly lower than that of the control group, throughout the entire aging process (*p* < 0.05), except at 24 h. In the NAC group, the CAT activity increased at 2–12 h (*p* < 0.05) and was 5.47% and 16.42% higher than those in the control and H_2_O_2_ groups at 12 h, respectively. The SOD activity in the NAC group at 24 h had 49.72% and 27.7% higher activity than the control and H_2_O_2_ groups (Figure 1C). In results consistent with the present findings, Shieh et al. [29] demonstrated that NAC effectively inhibited mitochondrial ROS production and reversed the decrease in antioxidant enzyme activity. However, SOD and GSH-Px activities (Figure 1D) in the H_2_O_2_ group were significantly lower than those in the control and NAC groups (*p* < 0.05), except at 24 h and 6 h. The present results were also supported by reports in [19], which showed that oxidative stress induced by H_2_O_2_ decreased the activities of antioxidant enzymes and increased ROS production.

ROS damage the mitochondrial membrane by oxidizing polyunsaturated fatty acids. As a result, MDA production and the MPTP opening increase can indirectly reflect the degree of oxidative stress induced by ROS [30,31]. The MDA level in the H_2_O_2_ group significantly increased by 35.43% from 2 to 12 h, and it was much higher than that in the control and NAC groups during storage, except at 2 h (*p* < 0.05; Figure 1E). Meanwhile, MDA was inhibited in the NAC group and reached a minimum value at 24 h. These results indicate that the ROS overload in mitochondria was induced by H_2_O_2_. In addition, ROS-mediated oxidative damage to mitochondria increased the MDA content but decreased the activities of antioxidant enzymes, implying that ROS induced by oxidative stress promoted mitochondrial damage.

### 3.2. Changes in Mitochondrial Dysfunction

**Figure 2:**
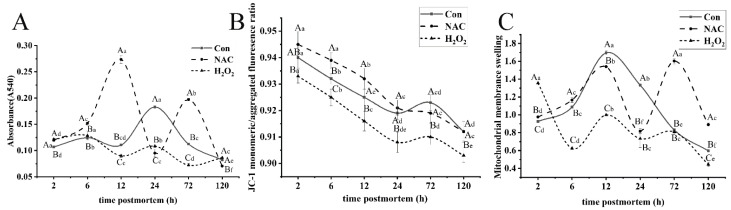
Changes in mitochondrial membrane opening (**A**), MMP (**B**), and mitochondrial swelling (**C**) of fish muscle during postmortem storage in the NAC, H_2_O_2_ and control groups. The lowercase letters represent the differences in the three groups at different times (*p* < 0.05). The differences among the three groups at the same time point were calculated via Duncan’s multiple-range test, represented by capital letters. The error bars indicate the standard error of the mean (*n* = 3).

MPTP is an important protein in the architecture of mitochondrial membrane channels. The opening of MPTP can increase the permeability of the inner membrane of mitochondria, leading to mitochondrial dysfunction, such as mitochondrial swelling and the collapse of mitochondrial membrane potential [11]. In order to further investigate the role of oxidative stress in the mitochondria of fish muscle, the MPTP opening and MMP were examined. As shown in Figure 2A, significant differences in the MPTP opening levels were found in the control, NAC, and H_2_O_2_ groups in postmortem fish muscle (*p* < 0.05). The MPTP opening decreased significantly at 2–24 h, and the optical density reached the maximum at 24 h in the control group. Conversely, the absorbance was markedly lower for samples incubated with NAC, especially at 12 h, indicating that MPTP opening occurred at the early stages of aging. MPTP opening in the H_2_O_2_ group increased by 40.38%, from 2 to 72 h, and its opening was significantly greater than that in the control group at 12–72 h (*p* < 0.05). This suggests that H_2_O_2_ enhanced the MPTP opening by inducing mitochondrial oxidative damage. Based on Figure 2B, MMP decreased sharply among the three groups from 2 to 24 h and decreased slightly after 24 h (*p* < 0.05). The percent decrease from 2 to 24 h was 2.28%, 2.61% and 2.75%, respectively, in the control, NAC and H_2_O_2_ groups. Moreover, the MMP in the H_2_O_2_ group was much lower than those of the NAC and control groups (*p* < 0.05). These results indicate that oxidative stress can influence the depolarization of MMP, which was observed most notably in the early stages of aging. ROS generation may influence the MPTP opening and MMP, which likely affect the mitochondrial apoptotic pathway [24].

Excessive mitochondrial swelling, which is strongly coupled with irreversible MPTP opening, is a crucial event that initiates mitochondrial-mediated apoptosis [32]. To better understand the extent of mitochondrial oxidative damage, the changes in mitochondrial swelling of the three treatment groups during postmortem aging were investigated (Figure 2C). Mitochondrial swelling significantly increased by 64.83% after 12 h postmortem (*p* < 0.05) but decreased from 0 to 12 h in samples with no treatments. The trend is characterized by a marked increase in mitochondrial swelling before 6 h in the H_2_O_2_ group, which coincides with ROS-induced oxidative stress and promotes the activation of antioxidative enzyme activities at the early stage (Figure 1C,D). Mitochondrial swelling in the H_2_O_2_ group was much higher than that in the control and NAC groups, except at 2 h and 72 h (*p* < 0.05). Thus, oxidative stress in the postmortem process inevitably causes mitochondrial swelling and dysfunction. Although the scavenging of ROS is mediated by antioxidant enzymes in muscles, ROS overload in cells induces mitochondrial dysfunction, leading to the activation of mitochondrial apoptosis during postmortem storage [33].

### 3.3. ROS Promoted Cytochrome C Expression and Oxidative Redox Level

**Figure 3:**
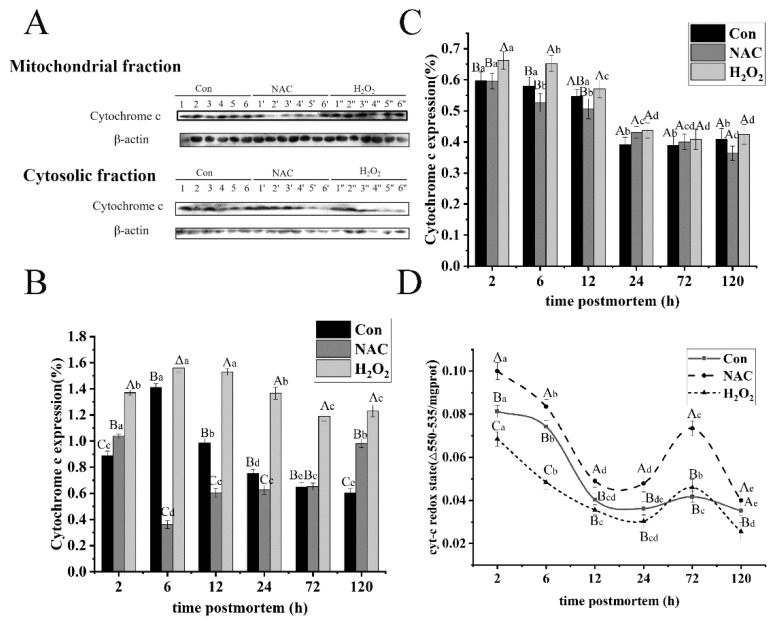
Representative Western blots (**A**) and expression level of cytochrome c (**B**–**D**) in both mitochondria and cytoplasm fractions of fish muscle obtained at 2–120 h of postmortem storage. β-actin was used as the loading control. The lowercase letters represent the differences in the three groups at different times (*p* < 0.05). As such, 1, 2, 3, 4, 5 and 6; 1′, 2′, 3′, 4′, 5′ and 6′; 1″, 2″, 3″, 4″, 5″ and 6″ represent samples stored for 2, 6, 12, 24, 72 and 120 h at 4 °C in the control, NAC and H_2_O_2_ treatment groups, respectively. The differences among the three groups at the same time point were determined by Duncan’s multiple-range test, represented by capital letters. The error bars indicate the standard error of the mean (*n* = 3).

Cytochrome c was released from mitochondria into the cytoplasm, as a key initiating event triggered in caspase activation during apoptosis [34]. To evaluate whether cytochrome c release is due to ROS and its effect on the mitochondrial apoptotic cascade activation, the expressions of cytochrome c, in both the mitochondria and cytoplasm, were determined through Western blot analysis (Figure 3A). In the mitochondrial fraction (Figure 3B), the abundance of cytochrome c in the control group increased by 37.09% from 2 to 6 h, and then decreased as postmortem aging progressed (*p* < 0.05). The findings may be due to the increase in active cytochrome c levels. Moreover, the release rate of cytochrome c was lower than its production in the mitochondrial fraction, similar to the reports by Huang et al. [34]. The amount of cytochrome c in the H_2_O_2_ group was much higher than that in the control and NAC groups, over the entire aging period (*p* < 0.05). Additionally, cytochrome c expression was characterized by a decline during the first 6 h (*p* < 0.05) and a slight increase from 12 to 72 h in the NAC group (Figure 3B). The results imply that ROS formation induced by H_2_O_2_ can stimulate an increase in cytochrome c in mitochondria, and the cytochrome c is released to the cytoplasm in the early phase of postmortem, which was consistent with the report of Wang et al. [19].

In the cytoplasm fraction, cytochrome c expression (Figure 3C) decreased by 34.65%, 27.71% and 34.04% from 2 to 24 h in the control, NAC and H_2_O_2_ groups, respectively. A decrease in cytochrome c may act as an ROS scavenger against oxidative stress. The cytochrome c level was much higher in the H_2_O_2_ group than in the control and NAC groups before 24 h (*p* < 0.05). However, no changes were observed among the three different treatments after 12 h (*p* > 0.05). These results are consistent with the idea that ROS generation, exerted by oxidative stress, accelerates the release of cytochrome c from mitochondria to the cytoplasm, which is, in turn, mediated by some pro-apoptotic factors, accompanied by mitochondrial damage (Figure 1). These findings were consistent with those reported by Wang et al. [19], indicating that ROS promoted greater mitochondrial damage, stimulating the apoptotic process via cytochrome c release.

The redox state of cytochrome c was considered a possible factor in the mediation of the mitochondrial apoptotic pathway. Moreover, it has been demonstrated that only oxidized cytochrome c formation is capable of inducing the apoptotic-process-activating caspases [35]. The reduction state of cytochrome c is less effective in mediating the formation of apoptosomes and the activation of the caspase cascade [19]. Thus, as far as the changes in the redox state of cytochrome c were concerned, the finding is presented in Figure 3D. A significant decile of the reduction state of cytochrome c was observed before 72 h with 55.44%, 52.12% and 55.54% in the control, NAC and H_2_O_2_ groups, respectively. This was consistent with a report indicating that the oxidation level of cytochrome c increased from 2 to 72 h postmortem, thus, mediating apoptosis [9]. In addition, the reduction level of cytochrome c in the control group was lower than that in the NAC group, throughout the entire aging process (*p* < 0.05). By contrast, compared with the H_2_O_2_ group, its redox state was much lower than that of the control and NAC groups during postmortem storage (*p* < 0.05). These outcomes indicate that a significantly higher cytochrome c oxidation level during the aging period may be attributed to oxidative stress in mitochondria, leading to the mediation of caspase-3 activation.

### 3.4. Mitochondrial Morphology

**Figure 4:**
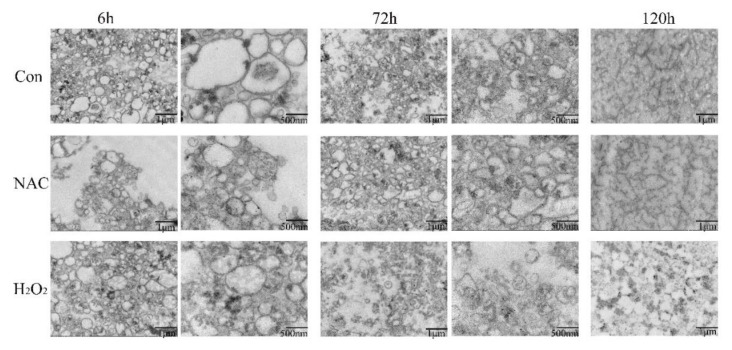
Transmission electron microscopy analysis of mitochondrial ultrastructure of fish muscle at 6, 72 and 120 h. Scale bar: 1 μm and 500 nm.

Isolated mitochondria showed morphological changes from an ultrastructural perspective, as visualized by TEM (Figure 4). The changes in mitochondrial damage as postmortem aging progressed were evaluated. Swollen mitochondria and large vacuoles appeared at 6 h postmortem with no treatments. It was not in agreement with the reports of Yu et al. [26], due to the different species. As storage time increased, mitochondria separated, demonstrating more apparent morphological damage. The mitochondria exhibited vague outer membranes and cristae, as well as an increase in vacuoles. Mitochondria in the NAC treatment at 6 h exhibited a greater intact mitochondrial structure, and fewer mitochondria were destroyed, compared with the control group. Conversely, at the same time, the H_2_O_2_ group exhibited mitochondrial swelling and vague cristae fracture. At 3 d postmortem, the mitochondrial profiles in the NAC group were clearer and more intact than those in the control group, implying that NAC treatment alleviated mitochondrial damage and prevented mitochondrial dysfunction during postmortem storage. In particular, disorganized mitochondrial crests and intermembrane separation occurred in the H_2_O_2_ group after 3 d, which provided direct evidence of oxidant-exaggerated mitochondrial damage by ROS-induced oxidant stress. Moreover, at 5 d postmortem, significant mitochondrial dysfunction was apparent due to fragmented morphology. Additionally, the outer membranes in isolated mitochondria showed significant injury, especially in the H_2_O_2_ group. When oxidative stress exceeds the adaptive capacity of the cell, the lower intracellular levels of ATP may lead to cell apoptosis, accompanied by mitochondrial dysfunction, extensive mitochondrial fragmentation, and the release of cytochrome c [36].

### 3.5. Effect of ROS-Mediated Structural Protein Degradation

#### 3.5.1. Titin and Nebulin

**Figure 5:**
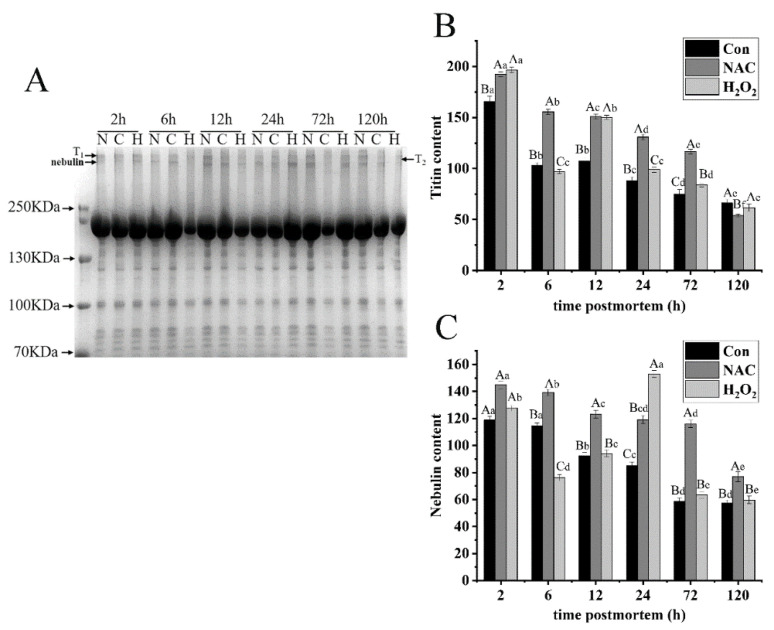
Representative SDS-PAGE (**A**) of protein degradation in fish muscle during postmortem storage. The degradation of titin (**B**) and nebulin (**C**) was studied in the NAC, H_2_O_2_ and control groups. Sixty micrograms of proteins was loaded per lane. T1 refers to intact titin, and T2 refers to the large degradation products from intact titin. The lowercase letters represent the differences in the three groups at different times (*p* < 0.05).

Titin is a giant elastic protein (3000 kDa) connecting the Z-disk and M-band regions of the sarcomere. It is largely responsible for the passive stress that develops when muscles are stretched [37]. Because titin is susceptible to degradation, it primarily displays double bands during the early stages of postmortem aging. The upper band is referred to as T_1_, whereas the lower band is referred to as T_2_, the degradation product of T_1_. After 2 h of aging, titin exhibited a mixture of bands, T_1_ and T_2_, among the three different treatments. A significant decline in titin, by 71.92% and 59.99%, was observed in the NAC and control groups during aging, respectively (*p* < 0.05; Figure 5A,B), and the amount of titin in the control group was lower than that in the NAC group, except at 120 h (*p* < 0.05). This implies that titin in fish is susceptible to degradation, and NAC treatment could effectively mitigate its degradation. The T_2_ band was the prominent band after 6 h postmortem storage in the control group. This result is in accordance with our previous study [10], where a degradation product (T_2_) was observed in *Esox lucius* myofibrils at the early stages of aging. In addition, titin content decreased by 37.73% from 2 to 6 h but increased from 6 to 12 h, relative to the H_2_O_2_ group, and its level was much higher than that in the control group from 12 to 72 h. This might be the result of protein cross linking, induced by H_2_O_2_, which hindered titin degradation.

Nebulin, a large protein (800 kDa) in skeletal muscles, constitutes a series of filaments anchored at the Z-line [38]. No changes in nebulin were observed in the control group from 2 to 6 h, but a significant decrease of 49.84% was observed from 6 to 120 h postmortem (*p* < 0.05; Figure 5C). This implies that nebulin exhibited relative integrity at the early stage of aging. Over the entire storage period, nebulin degradation in the H_2_O_2_ group was notably higher than that in the control and NAC groups (*p* < 0.05), except at 2 h. A significant decrease was observed in the H_2_O_2_ group from 2 to 6 h, and a sharp increase from 6 to 24 h (*p* < 0.05) was due to the role of oxidation, caused by H_2_O_2_. At 24 h, a new band of nebulin degradation was found in the H_2_O_2_ group. In addition, the band intensity of nebulin and titin in the control group gradually decreased during the entire storage period. Nebulin and titin decreased by as much as 28.53% and 32.76% on the first day, respectively. Troponin-T decreased by 24.49% from 2 to 24 h, supporting the idea that large proteins are more susceptible to degradation during postmortem storage. The combined results suggest that oxidation may change the structure of large myofibrillar proteins, thereby inducing their aggregation from the very beginning.

#### 3.5.2. Actin

**Figure 6:**
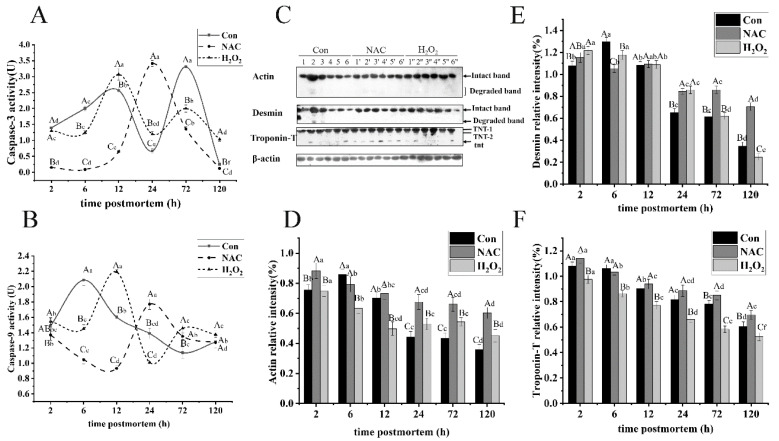
The analysis of caspase-3 and caspase-9 activities (**A**,**B**) and representative Western blots (**C**) of actin, desmin and troponin-T in fish muscle after slaughter from the NAC, H_2_O_2_ and control groups. The degradation of myofibrils (actin, desmin, and troponin-T) was determined during postmortem storage (**D**–**F**). As such, 1, 2, 3, 4, 5 and 6; 1′, 2′, 3′, 4′, 5′ and 6′; 1″, 2″, 3″, 4″, 5″ and 6″ represent samples stored for 2, 6, 12, 24, 72 and 120 h at 4 °C in the control, NAC and H_2_O_2_ treatment groups, respectively. Approximately 10 μL proteins was loaded per lane, and β-actin was used as the loading control. The lowercase letters represent the differences in the three groups at different times (*p* < 0.05).

Actin is recognized as the main component in the thin filaments in the sarcomere, and it is a good marker for tenderization in meats [39]. As shown in Figure 6C,D, the degradation product was first detected at 6 h in the control group but not in the H_2_O_2_ and NAC groups. The degradation levels of actin with H_2_O_2_ treatment significantly increased during storage (*p* < 0.05), because the methionine oxidation destroyed the non-covalent interactions in actin and reduced the stability of actin. This result is in agreement with a study by Liu et al. [40]. The authors found that actin content gradually decreased in the presence of an oxidant, which may be related to enzymatic oxidation. However, no obvious actin degradation was observed from 24 to 72 h in the H_2_O_2_ treatment group (*p* > 0.05). The level of actin in the skeletal muscles of the NAC group was significantly higher than that in the skeletal muscles of the control group (*p* < 0.05). However, actin content decreased by 22.65%, 28.55%, and 50.85% at 24, 72, and 120 h postmortem, respectively. The results indicate that NAC could prevent actin from proteolysis and preserve the intact structure of thin filaments. Similarly, the degradation of actin after oxidant treatment was significantly inhibited from 24 to 120 h, compared to the control group (*p* < 0.05). An increased oxidant also decreased the sensitivity of actin to caspase-3 proteolysis.

#### 3.5.3. Desmin

Desmin is the main intermediate filament protein that links adjacent myofibrils. Its proteolysis would cause the loss of transversal alignment of the sarcomeres and the weakening of the ordered and overall structure of muscles [41]. Similar to the degradation of actin, the degradation product of desmin was first detected at 6 h postmortem in the control group (Figure 6C). The expression of intact desmin in the H_2_O_2_ group gradually decreased with increasing storage time, and it declined by as much as 79.75% on day 5 (*p* < 0.05, Figure 6E). However, the almost 67.81% and 38.84% desmin were broken down overall during the postmortem storage in the control group and incubation with NAC, respectively. The results indicate that oxidation increased the susceptibility of desmin to degradation via caspase-3. However, desmin degradation in the NAC treatment group was significantly inhibited from 24 to 120 h, compared with the control group (*p* < 0.05). Desmin degradation in the H_2_O_2_ group significantly increased after 12 h (*p* < 0.05). This observation is supported by the findings in [42]. The authors demonstrated that moderate oxidation promoted the degradation of desmin. Moreover, its hydrolysis occurred early during postmortem aging. This is likely caused by the increasing oxidative level, which promotes the cleavage of the desmin structure and changes its susceptibility to μ-calpain and caspase-3 [43]. Interestingly, desmin and titin in the control group exhibited higher proteolysis rates than other myofibrillar structure proteins, with decreases in content by 39.35% and 32.76% on the first day, respectively.

#### 3.5.4. Troponin-T

Troponin-T is part of the troponin complex and is identified as a marker of ongoing proteolysis. Bands of 30-kDa fragments, derived from intact troponin-T, are closely related to meat tenderness. As shown in Figure 6C, two isoforms of troponin-T (TNT-1, TNT-2) and 30-kDa band (tnt) in fish muscle were observed, and these observations are consistent with a previous report by Ding et al. [44]. The tnt was detected in the NAC group at slaughter. Its degradation level gradually increased from 2 to 24 h and then decreased, suggesting that NAC does not affect μ-calpain activity. Additionally, the intact troponin-T in the NAC group was higher than that in the control group from 24 to 72 h (*p* < 0.05; Figure 6F). On the contrary, intact troponin-T significantly decreased by 43.95%, 39.01%, and 46.36% during postmortem storage in the control, NAC, and H_2_O_2_ groups, respectively. The intact troponin-T in the H_2_O_2_ group was lower than that in the control group (*p* < 0.05). Collectively, these results show that oxidation induced the degradation of troponin-T, due to the activation of caspase-3 by H_2_O_2_. Different results were reported, which found that the degradation of troponin-T in the sample incubated with H_2_O_2_ was hindered [43]. This effect could be explained by differences in species and treatments. Oxidation may enhance the activity of enzymes, such as caspase-3 and μ-calpain, and promote the degradation of troponin-T.

### 3.6. Changes in Caspase Activity during Postmortem Storage

The caspase cascade is thought to be crucial to mitochondrial apoptosis, initiated through the assembly of multiprotein complexes, which trigger the activation of initiator caspases. Then, the release of initiator caspases activate the effective caspase downstream [45]. Therefore, we explored the effect of oxidative stress induced by ROS on caspase-3 activation during postmortem storage. Caspase-3 and caspase-9 activities in fish were investigated, as shown in Figure 5. The caspase-9 activity in the control group increased by 28.53% from 2 to 6 h but significantly decreased after 6 h. Their activities were much lower from 24 to 72 h, relative to the NAC group (*p* < 0.05). Conversely, the caspase-9 activity in the H_2_O_2_ group reached the maximum at 12 h and was much higher than that in the skeletal muscles of the control group at 12, 72 and 120 h (*p* < 0.05; Figure 5A). Caspase-3, as the most significant effector caspase in cell apoptosis, plays a key role in proteolysis [46]. In the NAC group, the caspase-3 activity significantly increased from 2 to 24 h and peaked at 24 h. However, the activity was much lower than that in the control group, except at 24 h (*p* < 0.05). The H_2_O_2_ group showed higher caspase-3 activity at 12, 24, and 120 h, compared with the control group, especially at 12 h postmortem (*p* < 0.05; Figure 5B). This occurrence disagreed with previous reports [19,47]. The authors demonstrated that caspase-3 activity peaked at 24 h in the H_2_O_2_ group, and no significant changes were observed for caspase-9 activity during the entire postmortem storage, except at 24 h. The phenomenon possibly resulted from variations among fish, yak and goose meats. The above results show that ROS-induced oxidative stress promotes caspase activation, and the downstream effector caspase-3 might be activated by caspase-9. Collectively, the data indicate that oxidative stress leads to mitochondrial damage, a decrease in antioxidant enzyme activities, and the release of cytochrome c, which, ultimately, contribute to the activation of caspase-3.

## 4. Conclusions

Oxidation induced by ROS accelerated mitochondrial oxidative stress, leading to a decrease in antioxidative enzyme activities (CAT, SOD and GSH-Px) and an increase in MDA concentration. Oxidative stress may be generated and mediated by ROS, which increased MPTP opening, mitochondrial membrane swelling, and MMP collapse during postmortem storage. Enhanced generation and translocation of cytochrome c in oxidized fish muscle also induced caspase-3 activation by initiating the mitochondrial apoptotic pathway. Moreover, oxidation caused the degradation of small proteins but hindered the degradation of large proteins. This is because oxidation induced changes in myofibrils, resulting in different susceptibilities to proteolysis. These observations indicated that oxidative stress activates mitochondrial apoptosis, involved in regulating the changes in apoptotic factors in fish muscle, and contributes to structure proteins proteolysis. The mechanism involved in the oxidation of fish meat texture is complex, particularly the modification of myofibrillar proteins and the regulation of mitochondrial apoptotic factors.

## Data Availability

The data presented in this study are available on request from the corresponding author.
